# Pollution Characteristics, Sources, and Health Risks of Organochlorine Pesticides and Polychlorinated Biphenyls in Oviductus Ranae from Northern China

**DOI:** 10.3390/toxics14010101

**Published:** 2026-01-21

**Authors:** Shizhan Tang, Haonan Zhang, Peng Wang, Dongli Qin, Zhongxiang Chen, Guo Hu

**Affiliations:** 1 Heilongjiang River Fisheries Research Institute, Chinese Academy of Fishery Sciences, Harbin 150070, China; tangshizhan@hrfri.ac.cn (S.T.); zhanghaonan0524@163.com (H.Z.); wangpeng@hrfri.ac.cn (P.W.); qindongli@hrfri.ac.cn (D.Q.); 2 Supervision, Inspection and Testing Center for Fishery Environment and Aquatic Products (Harbin), Ministry of Agriculture and Rural Affairs, Harbin 150070, China; 3 College of Fisheries and Life Science, Shanghai Ocean University, Shanghai 201306, China

**Keywords:** Oviductus Ranae, OCPs, PCBs, pollution characteristics, health risk

## Abstract

This study systematically analyzed the pollution levels, distribution characteristics, and associated health risks of 17 organochlorine pesticides (OCPs) and 9 polychlorinated biphenyls (PCBs) in Oviductus Ranae (*Rana dybowskii*) from major production areas in Heilongjiang Province, China. OCPs and PCBs were detected in all samples. The total concentration of OCPs ranged from 11.7 to 67.9 ng/g (dry weight), while that of total PCBs ranged from 4.43 to 8.06 ng/g. Endosulfans constituted the predominant OCP group, accounting for 54.5% of ∑OCPs, with an α/β-endosulfan ratio (~2:1) indicative of recent agricultural input. Among DDTs, the dominance of p,p′-DDE and the absence of parent DDT suggested aerobic degradation of historical residues. For HCHs, the isomer profile (β-HCH predominance, α/γ-HCH = 0.27) pointed to weathered lindane sources. The PCB profile was uniquely dominated by lower-chlorinated congeners (PCB1 and PCB29), implying influences from atmospheric transport and/or in situ microbial dechlorination of legacy PCBs. The persistent organic pollutants (POPs) contamination profile in Oviductus Ranae reflects a combined influence of recent pesticide application, weathered historical residues, and long-range transport. Although the concentrations are below current regulatory limits, the cumulative and persistent nature of these POPs, coupled with the product’s medicinal use, justifies a precautionary stance regarding long-term consumption. The distinct congener patterns underscore the necessity for future research to prioritize the environmental behavior and toxicology of dominant transformation products within such specific agro-ecosystems.

## 1. Introduction

The brown frog (*Rana dybowskii*) is an ecologically and culturally significant species in the forest and aquatic ecosystems of Heilongjiang Province in northeastern China. The processed oviducts of female frogs, known as Oviductus Ranae, represent a valued traditional Chinese medicinal material with a long history of use and significant economic importance [1,2]. The promotion of the “pond culture—forest grazing relay model” relay farming model and the classification of *R. dybowskii* as an aquatic animal for management have led to a continuous expansion of its farming scale [1]. However, as the frog’s life cycle spans both aquatic and terrestrial environments and involves various human interventions during farming, these animals may be exposed to and bioaccumulate multiple contaminants such as antibiotics, heavy metals, microplastics, and persistent organic pollutants (POPs) through various pathways. Consequently, concerns regarding the quality and safety of this product are growing [3,4,5].

Polychlorinated biphenyls (PCBs) and organochlorine pesticides (OCPs) are listed as POPs under the Stockholm Convention due to their high persistence, toxicity, and bioaccumulative potential, and have been progressively banned worldwide [6,7,8]. Nevertheless, their residues persist in various ecosystems owing to their high stability and sorption capacity. Notably, forest ecosystems are recognized as significant reservoirs for POPs, primarily because atmospheric POPs can be captured by forest canopies, transferred to soils, and subsequently accumulated and stored in this organic carbon-rich medium [9,10]. Given their important role within forest ecosystems, *R. dybowskii* faces evident exposure risks to POPs; however, related research remains scarce. Heilongjiang Province, serving as a crucial ecological barrier and major forestry region in northeastern China, historically employed POPs such as OCPs and PCBs extensively. Studies have reported detectable concentrations of OCPs and PCBs in soil and water systems within this region, with DDTs, hexachlorocyclohexanes (HCHs), hexachlorobenzene (HCB), and PCBs being the primary contaminants, exhibiting a 100% detection rate. These pollutants readily accumulate in adipose tissues through bioaccumulation and biomagnification processes, posing potential health risks to humans [11]. Furthermore, frequent forest fires in this region can remobilize persistent organic pollutants (POPs) from soils and vegetation into the environment [12,13]. For *R. dybowskii*, which is farmed under a “pond culture—forest grazing relay model”, the forest-release stage involves direct habitation on the forest floor. Consequently, the re-emission and subsequent deposition of POPs induced by wildfires may represent a significant exposure pathway for these frogs, either through direct inhalation/dermal contact or via the ingestion of contaminated forest-floor invertebrates (e.g., insects). Globally, PCBs and OCPs, both in environmental media and biota, are frequently reported at concentrations ranging from ppt (ng/L or ng/kg) to ppb (μg/L or μg/kg) levels [14,15,16]. Additionally, studies have found that PCBs (Aroclor 1248) exhibit high embryotoxicity to *R. dybowskii* and inhibit oocyte maturation [17]. Research on PM2.5-bound PCBs and OCPs in rural northern China identified waste incineration and industrial activities as sources of PCBs, while DDTs and HCHs originated primarily from pesticide use. PCB exposure was at a moderate level, posing potential risks to humans [5]. Another study on POP bioaccumulation in loach (*Misgurnus anguillicaudatus*) from northeastern China found relatively high contamination levels of DDTs, HCHs, and HCB, with significant carcinogenic risks associated with consuming loach from this region [15]. These findings indicate substantial research on POPs pollution in freshwater products and the environment of northeastern China. However, systematic studies on pollution characteristics, source apportionment, and risk assessment are notably lacking for amphibian-derived products. Among these, Oviductus Ranae—a valued traditional medicinal material produced under the distinctive “pond culture–forest grazing relay model”—represents a significant gap, having been largely overlooked in POP contamination and dietary risk assessment studies.

Therefore, this study aims to systematically analyze the pollution levels, compositional profiles, and spatial distribution patterns of OCPs and PCBs in Oviductus Ranae, and to assess the associated health risks from oral exposure. The findings are expected to provide a direct scientific basis for establishing food safety standards specific to this product, guiding environmental management practices in its farming habitats, and supporting the sustainable development of regional amphibian resource utilization.

## 2. Materials and Methods

### 2.1. Study Area and Sample Collection

This study selected six major R. dybowskii farming areas in Heilongjiang Province. A total of 1080 adult female frogs were collected from 54 sites. The specimens were collected between September and October, coinciding with the period when frogs return from the forest to their hibernation habitats at lower elevations. Each farming area comprised 9 sampling points (Figure S1). To obtain a representative average concentration and minimize the bias caused by individual variation, a composite sampling strategy was adopted. Specifically, 20 individuals of similar size (average body weight 40 ± 4.5 g) from each sampling point were pooled to form one composite sample. Prior to analysis, the oviductus ranae were lyophilized and stored in a desiccator at room temperature.

### 2.2. Instruments and Reagents

The instruments used included the 7000C-7890B Gas Chromatography-Tandem Mass Spectrometer (GC-MS/MS) equipped with an electron impact (EI) ion source (Agilent Technologies, Santa Clara, CA, USA), XS205 Dual Range Analytical Balance with a sensitivity of 0.01 mg (Mettler Toledo, Greifensee, ZH, Switzerland), Allegra X-30R High-Speed Centrifuge (Beckman Coulter, Brea, CA, USA), Microwave muffle furnace (Thermo Fisher Scientific, Waltham, MA, USA), N-EVAP112 Sample Concentrator (LWL, Shanghai, China), and Milli-Q Ultrapure Water System (Merck Millipore, Burlington, MA, USA).

To comprehensively investigate the potential sources of POPs contamination in Oviductus Ranae and the unique exposure pathways of amphibians in agro-ecosystems, this study expanded beyond conventional pollutant monitoring (covering 17 typical OCPs and 7 indicator PCBs) to include the detection of a terminal dechlorination product of PCBs (PCB1) and a historical legacy congener (PCB29). Mixed standard solutions of 9 PCBs and 17 OCPs at a concentration of 100 mg/L were purchased from Alta Scientific Co., Ltd. (Tianjin, China). n-Hexane, acetone, dichloromethane, and acetonitrile were all chromatographic grade reagents (J.T. Baker, Phillipsburg, NJ, USA). Solid-phase extraction (SPE) cartridges, including Floreisil, amino, and silica gel (500 mg/3 mL), were obtained from ANPEL Laboratory Technologies (Shanghai, China). Standard Solution Preparation: The mixed standard solution of 9 PCBs and 17 OCPs was diluted 100-fold to prepare an intermediate stock solution. Working standard solutions at concentrations of 1, 2, 5, 10, 20, 50, and 100 μg/L were then prepared in isooctane for subsequent analysis.

### 2.3. Analytical Methods

Chromatographic Conditions: HP-5MS capillary column (30 m × 0.25 mm × 0.25 μm); injector temperature: 290 °C; injection volume: 1 μL; carrier gas: high-purity helium (≥99.999%); flow rate: 1.0 mL/min; injection mode: splitless. The purge valve was opened at 1.0 min with a purge flow of 30 mL/min. The gas saver was activated at 2 min with a flow of 20 mL/min. Oven temperature program: initial temperature held at 60 °C for 1 min, increased to 120 °C at 30 °C/min and held for 1 min, then raised to 240 °C at 5 °C/min and held for 1 min, finally increased to 300 °C at 20 °C/min and held for 5 min. Mass Spectrometric Conditions: Electron impact (EI) ion source; ionization energy: 70 eV; ion source temperature: 300 °C; MS transfer line temperature: 280 °C; data acquisition mode: selected reaction monitoring (SRM); collision gas: high-purity helium (≥99.999%); solvent delay: 4.00 min. Sample Pretreatment: Briefly, 2.0 g of the prepared sample was weighed, and 100 μL of ^13^C_6_-HCB and ^13^C_12_-PCB52 (Sigma-Aldrich, St. Louis, MO, USA) at a concentration of 2.0 mg/L was added as an internal standard. Accelerated solvent extraction was performed for 10 min at 10.3 MPa using an n-hexane/acetone mixture (1:1, *v*/*v*). The crude extract was dried by passing it through a glass fiber filter containing anhydrous sodium sulfate (Sigma-Aldrich, Burlington, MA, USA), washed with an ethyl acetate-cyclohexane mixture (1:1, *v*/*v*), and filtered again. The combined extracts were concentrated by rotary vacuum evaporation at 40 °C. Sample cleanup involved passing the extract through a Florisil SPE cartridge (Agilent Mega BE-Fl type, Santa Clara, CA, USA), pre-conditioned with n-hexane, followed by elution with an ethyl acetate-cyclohexane mixture (1:1, *v*/*v*). The purified eluate was combined, dried under a gentle stream of nitrogen at 40 °C, reconstituted in 1 mL of isooctane, filtered through a 0.22 μm membrane, and then analyzed.

### 2.4. Quality Assurance and Quality Control (QA/QC)

Comprehensive quality assurance and control (QA/QC) procedures were implemented throughout the analytical process. The concentrations of target OCPs and PCBs were quantified using an internal standard method based on the relative response to the added ^13^C_6_-HCB and ^13^C_12_-PCB52. All calibration curves demonstrated excellent linearity, with correlation coefficients (R^2^) exceeding 0.998. The method detection limits (MDLs), defined at a signal-to-noise ratio of 3, ranged from 0.01 to 0.51 μg/kg for the target analytes (Table S1).

Procedural blanks were prepared using cod (*Gadus macrocephalus*) muscle tissue, which was selected due to its low background levels of the target POPs, homogeneity, and common use in food contaminant analysis. These blanks were processed alongside each batch of samples to monitor potential contamination. Method accuracy and precision, evaluated through matrix spike recovery tests, yielded recoveries of 87.6% to 108% with relative standard deviations (RSDs) of less than 15% (Table S1). Furthermore, replicate analyses were performed on approximately 10% of the samples to assess methodological reproducibility. All reported sample concentrations were blank-corrected.

### 2.5. Pollution and Health Risk Assessment

As *R. dybowskii* was only officially classified under fishery management as an aquatic animal in China in 2020, specific quality standards for this product remain underdeveloped. Therefore, to comprehensively evaluate its pollution status, we referred to multiple relevant Chinese regulatory standards. For each target pollutant, the most stringent maximum limit available across these standards was applied to ensure a conservative and health-protective assessment. This study selected HCHs, DDTs, and PCBs, referencing corresponding Chinese regulatory limits, and employed the single-factor pollution index method for assessment:

(1) $$P_{i}=\frac{C_{i}}{C_{si}}$$

where *P_i_* is the pollution index for the *i*-th pollutant in the Oviductus Ranae; *C_i_* is the measured concentration of the *i*-th pollutant (mg/kg dry weight); and *C_si_* is the reference standards for the *i*-th pollutant (mg/kg). Reference standards were adopted from NY 5073-2006 (Maximum limit of toxic and hazardous substances in pollution–free aquatic products) [18], NY/T 1516-2020 (Green food–Frog and its processed products) [19], GB/T 19507-2008 (Product of geographical indication-Jilin Changbaishan forest frog’s oviduct) [20], and GB 2762-2022 (National food safety standard–Maximum levels of contaminants in foods) for HCHs, DDTs, and PCBs [21]. The pollution level was categorized as follows [1]:

*P_i_* < 0.2: Background level (unpolluted)
0.2 ≤ *P_i_* < 0.6: Mild pollution
0.6 ≤ *P_i_* < 1.0: Moderate pollution
*P_i_* ≥ 1.0: Severe pollution

### 2.6. Health Risk Assessment

The estimated daily intake (*EDI*, mg/kg/Day) of OCPs depends on the concentrations of OCPs in each Oviductus Ranae and the amount consumed daily. In the following formula, *MC* (mg/kg, dry weight) is the detected concentration of each OCPs in the Oviductus Ranae, and *W* (kg/day) refers to the daily consumption weight of Oviductus Ranae, which is estimated by the daily consumption of aquatic products in China at 3 g/day [1]. The average weight (*AW*) of Chinese adults is 61.75 kg.

(2) $$EDI=\frac{MC\times W}{AW}$$

The target hazard quotient (*THQ_i_*) provides a quantitative standard to measure the exposure risk of OCPs. *EFr* is the exposure frequency (365 days/year). *ED* is the exposure duration which can last 70 years on average. *RfD* is the daily oral reference dose, and the average exposure time for non-carcinogens (*ATn*) equals *ED* × 365 days. A *THQ* value of <1 indicates a low or acceptable risk from dietary exposure, whereas a *THQ* ≥ 1 indicates a potential health concern that warrants further attention [1].

(3) $${THQ}_{i}=\frac{\mathrm{EFr}\times \mathrm{ED}\times \mathrm{EDI}}{RfD\times ATn}\times 10^{-3}$$

*TTHQ* (total target hazard quotient) is the sum of *THQ* of the OCPs to assess the dietary risk of Oviductus Ranae.

(4) $$TTHQ=\sum_{i=1}^{n}{THQ}_{i}$$

In the Formula (4), *TTHQ* is the sum of *THQ* of OCPs, *TTHQ* ≤ 1.0, indicating no significant negative impact; *TTHQ* > 1.0, indicating possible negative impact on human health; when *TTHQ* is greater than 10.0, it indicates that there is a chronic toxic effect.

### 2.7. Statistical Analysis

Statistical analysis was conducted using SPSS Statistics 22.0 software. A one-way ANOVA (with Tukey post hoc test) was employed to test the differences between different groups. For data that did not meet the normal distribution criteria, a nonparametric Kruskal–Wallis test and its post hoc test were applied. In cases where the percentage of detection data below the limit of detection (LOD) was less than 60%, all results below the LOD were calculated as 1/2 LOD. This substitution method is widely adopted in environmental contaminant studies as it provides a conservative yet reasonable estimate that minimizes bias in summary statistics (e.g., means, correlations), compared to using zero or the full LOD value. To assess the correlations and group the OCPs and PCBs contents in Oviductus Ranae from different Sampling regions, Pearson correlation analysis and principal component analysis (PCA) were applied. A *p*-value less than 0.05 was considered statistically significant.

## 3. Results and Discussion

### 3.1. Pollution Levels and Distribution Characteristics

As shown in Table 1, the detection rates for OCPs and PCBs were 100% in all samples. The average concentration of ∑OCPs was 36.9 ng/g, and that of ∑PCBs was 5.85 ng/g. Endosulfans were the predominant OCPs, with an average concentration of 20.1 ng/g. The levels of α-endosulfan (12.3 ng/g) and β-endosulfan (6.88 ng/g) reflect the use patterns of technical endosulfan. The widespread detection of endosulfan sulfate (0.898 ng/g) indicates its environmental transformation and accumulation potential. Among DDTs, p,p′-DDE was the major metabolite, suggesting an aerobic degradation environment. β-HCH was the dominant isomer within HCHs, reflecting historical lindane usage and subsequent isomer transformation.

In this study, the observed diagnostic pattern strongly points to historical origins for the detected OCPs. Specifically, the complete absence of parent DDT compounds (o,p′-DDT and p,p′-DDT) and the sole detection of their metabolites (p,p′-DDD and p,p′-DDE) result in a (DDE + DDD)/∑DDTs ratio of 1. This, together with low α/β-HCH (0.12) and α-HCH/γ-HCH (0.26) ratios (Table 1), consistently indicates that the HCH and DDT residues primarily originate from historical applications rather than recent inputs [22,23,24]. Furthermore, the polychlorinated biphenyl (PCB) congener profile was dominated by lower-chlorinated congeners (PCB1 and PCB29), suggesting that the PCB composition in Oviductus Ranae reflects the environmental persistence and selective accumulation of these more volatile and mobile compounds. Their presence is likely associated with long-range atmospheric transport and legacy industrial sources [25].

**Figure 1:**
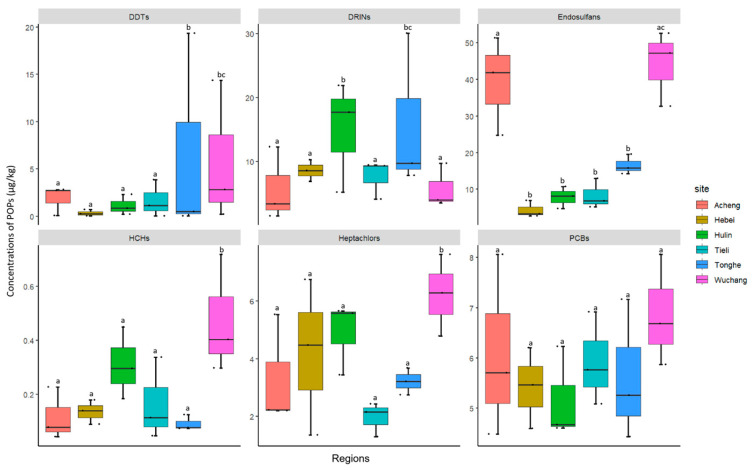
Concentrations of POPs (μg/kg dry weight) in Oviductus Ranae. Different lowercase letters (e.g., a, b, ab) above the bars indicate statistically significant differences among sampling sites for each pollutant, as determined by one-way ANOVA with Tukey’s post hoc test (*p* < 0.05). Sites sharing at least one common letter are not significantly different from each other.

Spatial distribution analysis (Figure 1) revealed significant differences (*p* < 0.05) in POP concentrations among sampling sites. Endosulfans and DDTs exhibited the most pronounced spatial heterogeneity, with significant concentration variations across sites (*p* < 0.05). This suggests that the sources and environmental fates of these pollutants may have strong local characteristics. In contrast, the distribution of PCBs was relatively uniform across sites, with no statistically significant differences detected (*p* > 0.05), indicating their sources are likely more related to regional-scale processes such as atmospheric deposition.

Regarding distribution hotspots, different pollutants exhibited distinct spatial aggregation patterns. The Wuchang area was the primary reservoir for HCHs and heptachlors, with concentrations significantly higher than those in Tieli and Hebei (*p* < 0.05). Acheng and Wuchang jointly constituted the distribution hotspot for endosulfans, with levels significantly exceeding all other sampling sites (*p* < 0.05). Tonghe exhibited unique DDT enrichment, with its average concentration significantly higher than areas like Acheng and Tieli (*p* < 0.05), establishing it as a distinct high-value zone for these pollutants. Consequently, this study employed the single-factor pollution index method to assess HCHs, DDTs, and PCBs against relevant Chinese regulatory limits. Although significant spatial variation in pollutant occurrence was observed, none reached pollution levels (Table S3).

This spatial differentiation pattern is likely closely related to local historical pesticide usage practices, land use patterns, and environmental geochemical characteristics. Wuchang and Acheng, as traditional agricultural regions, may have historically applied large quantities of endosulfans and HCHs, leading to higher soil residues. The high DDT residue in Tonghe may be associated with its specific industrial history or improper disposal of obsolete pesticides. The relatively uniform distribution of PCBs supports their characteristic as typical POPs subject to long-range transport, with sources likely more connected to regional atmospheric processes. A total of 209 polychlorinated biphenyl (PCB) congeners have been identified. Generally, lower-chlorinated PCBs exhibit greater environmental mobility and bioavailability, facilitating their entry into food webs and subsequent bioaccumulation, whereas higher-chlorinated PCBs tend to possess greater toxicity and environmental persistence [26]. Under anaerobic conditions, higher-chlorinated PCBs (e.g., penta- to hepta-chlorinated congeners such as PCB118, PCB138, and PCB153) can undergo microbial reductive dechlorination, progressively losing chlorine atoms [25]. PCB1 is a terminal product in certain dechlorination pathways, indicating that its detection in the environment likely results from the transformation of other, higher-chlorinated PCBs. Compared to PCB1, PCB29 has a moderate bioaccumulation potential. Historically, PCB29 was a minor component in early industrial PCB mixtures, used in dielectric fluids, hydraulic oils, plasticizers, and heat transfer fluids. It can also be a microbial dechlorination product of higher-chlorinated PCBs (e.g., PCB44) [27]. The concurrent detection of PCB1 and PCB29 in the Oviductus Ranae samples is consistent with suggests a scenario resulting from the combined effects of historical residues and environmental transport/transformation processes.

### 3.2. Correlation and Pollution Source Analysis of 17 OCPs and 9 PCBs in Oviductus Ranae

**Figure 2:**
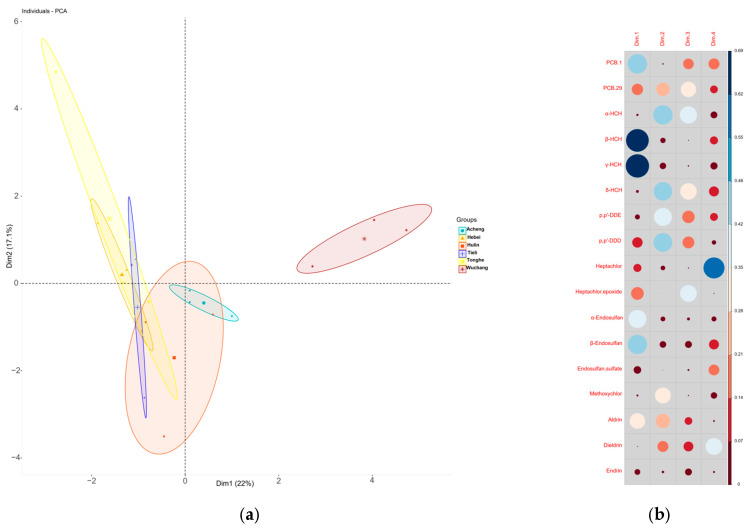

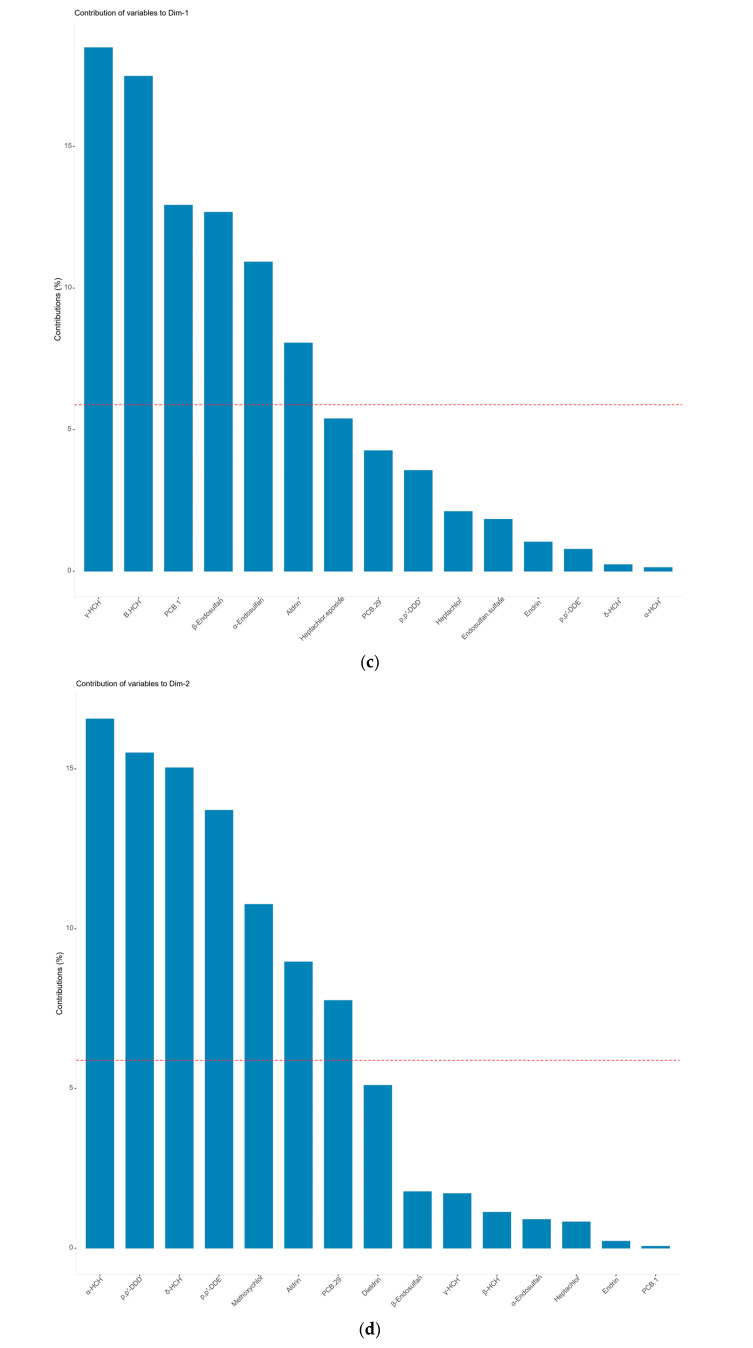
Principal component analysis (PCA). (**a**) Scatter plot scores of the first two principal components (PCs), (**b**) the representation of the first four major components as POPs, (**c**) variance interpretation rate of the first axis PCA, and (**d**) variance interpretation rate of the second axis PCA. Dim1 and Dim2 represent the first and second principal components, respectively, denoted as PC1 and PC2.

**Figure 3:**
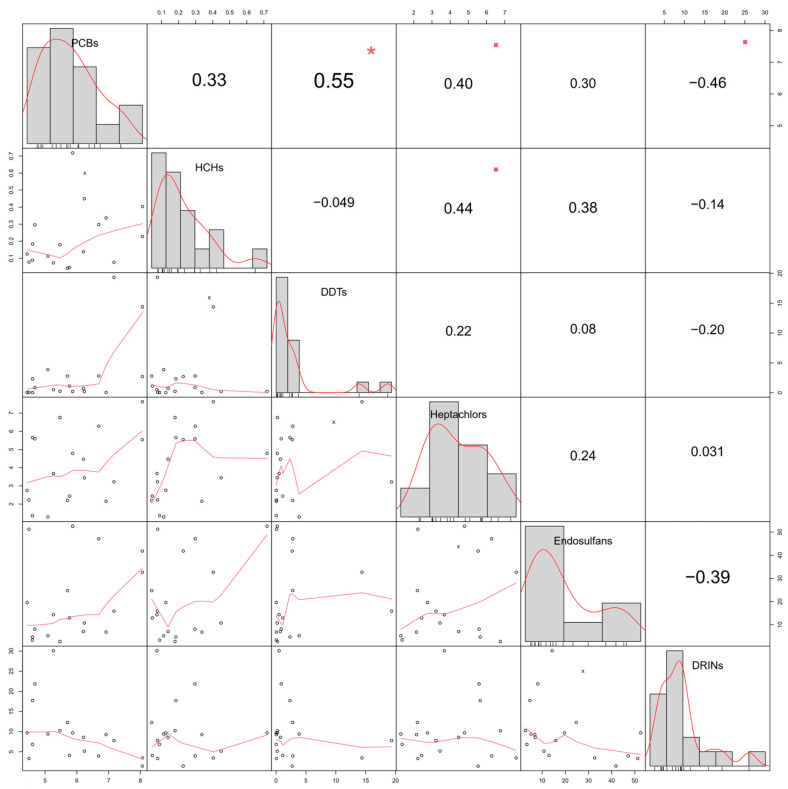
Spearman correlation coefficients among POPs in Oviductus Ranae. Asterisks (*) and squares are used to mark correlation coefficients that are statistically significant (*p* < 0.05).

To further investigate the source pathways and environmental behaviors of various POPs in Oviductus Ranae, principal component analysis (PCA) and correlation analysis were performed on the 17 OCPs and 9 PCBs (Figure 2 and Figure 3). For the principal component analysis, raw concentration data underwent a log_10_(x + 1) transformation to approximate a normal distribution, followed by standardization (z-score transformation) to remove scale differences among variables. PCA results showed that samples from different production areas exhibited some regional clustering along PC1 (22% variance) and PC2 (17.1% variance), indicating that geographical origin influences pollutant composition. Variable contribution analysis revealed that PC1 was primarily driven by p,p′-DDD, p,p′-DDE, heptachlors, and endosulfan sulfate, reflecting the predominance of historically used OCPs and their degradation products. PC2 was mainly associated with α-HCH, β-HCH, and some PCBs, indicating contributions from industrial-source compounds and atmospheric transport/input.

The correlation analysis of POPs in Oviductus Ranae revealed complex interactions among different contaminants, as well as potential differences in their sources and environmental behaviors, which are significant for understanding the transport and transformation of POPs in ecosystems. As shown in Figure 3, a significant positive correlation was observed between PCBs and DDTs (r = 0.55, *p* < 0.05), representing the strongest correlation among all contaminant pairs. This finding suggests that PCBs and DDTs may originate from common industrial or agricultural sources or have undergone similar physicochemical processes in the environment, such as long-range atmospheric transport or soil adsorption–desorption behavior, ultimately leading to their consistent accumulation trends in Oviductus Ranae [28,29]. It is well documented in other ecosystems that PCBs and DDTs, as typical POPs, frequently show correlated distribution and co-accumulation patterns, which aligns with the associations observed in the present study [26,30,31].

Further analysis indicated that PCBs also showed a significant positive correlation with heptachlors, but a significant negative correlation with DRINs. This divergence in correlation patterns may reflect differences in the degradation mechanisms of these contaminants in environmental media. Both PCBs and heptachlors are relatively stable compounds with strong environmental persistence and likely enter the food chain through similar pathways, leading to bioaccumulation [32]. The divergence in correlation between DRINs and PCBs may be associated with differences in their distribution within lipid-rich tissues or in their metabolic transformation pathways, which merits further investigation. Among other contaminant relationships, HCHs showed a significant positive correlation only with heptachlors, suggesting similarities in their sources or environmental fates. Both HCHs and heptachlors are historically widely used organochlorine pesticides with prolonged environmental persistence. For example, diagnostic ratios of HCHs and chlordanes (including heptachlors) in outdoor dust from Pakistan indicate their historical emissions and continued presence [33]. In Korean coastal waters, Japanese flying squid (*Todarodes pacificus*) have been used as bioindicators, with confirmed accumulation levels of POPs such as HCHs, DDTs, and chlordanes in their livers [34].

Notably, endosulfans exhibited generally weak correlations with other POPs (|r| < 0.39, *p* > 0.05). This result may reflect distinct differences in the usage history, application methods, or degradation characteristics of endosulfans compared to other POPs. Compared with other legacy POPs such as DDT and PCBs, endosulfan is generally considered to have a shorter environmental half-life [26,35]. Alternatively, its metabolic pathways in organisms may vary, leading to an independent accumulation pattern in Oviductus Ranae. For example, in fish from Lake Koka in Ethiopia, DDTs were the predominant pesticide residues, while the bioaccumulation pattern of endosulfans may differ [36].

In summary, PCA and correlation analysis not only statistically revealed the co-occurrence patterns and divergence trends of POPs in Oviductus Ranae but also provided important insights for identifying pollutant sources and elucidating their transport and transformation behaviors within environmental-biological systems. Particularly in the context of mixed contamination, positive correlations may indicate common source inputs or synergistic accumulation, while negative correlations suggest potential competitive mechanisms or source differences. These findings offer valuable scientific references for assessing the safety of Oviductus Ranae and formulating corresponding pollution control strategies.

### 3.3. Analysis of POPs Pollution Characteristics and Mechanisms in Oviductus Ranae

The bioaccumulation of persistent organic pollutants (POPs) in amphibians, particularly frogs and toads (*Bufo gargarizans*), is a critical environmental concern due to their ecological role as bioindicators and their sensitivity to environmental contaminants [37,38]. POPs are carbon-based chemical compounds characterized by persistence, resistance to degradation, potential for long-range environmental transport, and lipophilicity, enabling them to accumulate in the fatty tissues of organisms and persist in the environment for extended periods [39]. These properties contribute to their widespread global distribution and potential for bioaccumulation and biomagnification through food chains [38,39,40]. The pollution characteristics identified in this study for Oviductus Ranae show significant contrasts and connections with POPs accumulation mechanisms in aquatic organisms such as Pacific salmon. In the muscle tissue of pink salmon (*Oncorhynchus gorbuscha*) from the Sea of Okhotsk, OCP concentrations ranged from 22 to 56 ng/g wet weight, with HCH isomers constituting over 90% of the total pesticides [41]. This presents a sharp contrast to the endosulfan-dominated pollution profile observed in Oviductus Ranae. This divergence likely stems from differences in exposure pathways due to the amphibious nature of brown frogs: salmon primarily accumulate pollutants through the marine food web, whereas brown frogs accumulate OCPs during their terrestrial phase via the soil-invertebrate pathway. Notably, the salmon study also found that OCP concentrations in gonadal tissues were significantly higher than in muscle (∑OCPs in eggs reached 265–609 ng/g *w*/*w*) [41]. This aligns with the elevated pollutant levels found in Oviductus Ranae (an extract from reproductive tissue), indicating a tendency for POPs to accumulate in lipid-rich reproductive organs. Furthermore, the regional variation noted in the salmon study, which reported significantly higher POPs concentrations in fish from the Sea of Okhotsk than in those from the Poronai and Kamchatka river basins (*p* ≤ 0.05), parallels the elevated endosulfan levels observed in the Wuchang and Acheng regions in this study [41]. This similarity underscores the decisive influence of localized pollution sources (e.g., agricultural activities) on POPs accumulation in biota. Whereas this divergence highlights the fundamentally different exposure pathways between marine pelagic and terrestrial–aquatic systems; thus, the comparison serves to illustrate conceptual differences in POPs accumulation rather than to draw direct quantitative parallels.

Hexachlorocyclohexane (HCH) ranks among the most extensively used and commonly detected organochlorine pesticides in the environment. Owing to its relatively high volatility, HCH is prone to long-range atmospheric transport, facilitating its widespread distribution even into remote regions such as the Arctic. Certain HCH isomers are known to cause adverse effects on the central nervous, reproductive, and endocrine systems. While γ-HCH undergoes rapid metabolism, β-HCH often accumulates preferentially in lipid-rich tissues—including adipose tissue, blood, and breast milk—resulting in consistently higher residual concentrations. Conversely, α- and γ-HCH are typically the predominant isomers detected in environmental matrices such as soil, water, and air. The ratio of α- to γ-HCH is frequently utilized as a diagnostic indicator to trace the global dispersion and transport pathways of HCH residues [42]. The isomer-specific accumulation profile identified in Oviductus Ranae—characterized by the preferential retention of β-HCH and the predominance of p,p′-DDE—is further supported by findings from salmon studies, in which p,p′-DDE constituted the sole representative of DDT and its metabolites [41]. This selectivity likely stems from interspecies variations in enzymatic degradation efficiency. Specifically, the β-HCH isomer, owing to its enhanced structural stability, exhibits greater resistance to metabolic breakdown, thereby promoting its persistence and accumulation across diverse biological systems.

Regarding endosulfan metabolism, the accumulation of endosulfan sulfate observed in this study (average 0.898 ng/g) indicates the occurrence of biotransformation processes for endosulfan within brown frogs. This echoes the metabolic pattern observed for heptachlor: a Heptachlor epoxide/Heptachlor ratio of 2.25 demonstrates that the epoxidized metabolite is more prone to accumulation. Such metabolic transformations not only enhance the persistence of the pollutants but may also increase their toxicity, as endosulfan sulfate is considerably more toxic than its parent compounds. Amphibians are particularly vulnerable to POPs due to their permeable skin and biphasic life cycles, exposing them to contaminants in both aquatic and terrestrial environments [37]. Studies have demonstrated tissue-specific distribution of organic halogenated pollutants (OHPs, a type of POP) within frogs. For instance, in black-spotted frogs (*Rana nigromaculata*) collected from an e-waste-polluted area in South China, OHPs were detected in various tissues including muscle, liver, kidney, stomach, intestine, heart, and eggs [37]. The median total OHP concentration was significantly higher in the liver (1360 ng/g) compared to other tissues such as muscle (67.4 ng/g), kidney (277 ng/g), stomach (114 ng/g), intestine (67.8 ng/g), heart (29.2 ng/g), and eggs (23.9 ng/g) [28]. This differential distribution indicates the liver’s role as a major storage and detoxification organ for these pollutants. Maternal transfer of POPs is another significant aspect of bioaccumulation in amphibians. The transfer of POPs from mother frogs to their eggs has been observed, with a median total OHP concentration in eggs of 23.9 ng/g, representing about 10.3% of the corresponding maternal body burden [37]. This transfer can have substantial implications for offspring survival and development, as early life stages are often more susceptible to the toxic effects of environmental contaminants [37].

Notably, typical indicator PCBs (e.g., PCB-28, -52, -101, -138, -153, -180) were not detected in this study. This contrasts with the general understanding that these compounds are commonly detectable in environmental media across the Northern Hemisphere. This distinctive observation may be attributed to multiple factors: (1) amphibians may exhibit selective uptake and accumulation pathways for pollutants, preferentially accumulating more water-soluble and mobile lower-chlorinated congeners; (2) intensive farming practices (e.g., periodic pond drainage and cleaning) may alter the environmental fate and bioavailability of pollutants; and (3) historically deposited industrial PCBs in the region may have undergone long-term anaerobic microbial dechlorination, shifting the congener profile in environmental reservoirs toward lower-chlorinated products [25,26]. Future research should incorporate concurrent monitoring of farming environment matrices (water, sediment, soil) and other organisms within the food web to more comprehensively elucidate the transport, transformation, and bioaccumulation pathways of PCBs in this specific agro-ecosystem.

### 3.4. Risk Assessment of OCPs Intake from Oviductus Ranae

OCPs are known for their ubiquity, persistence, and bioaccumulation, posing significant threats to human health and biodiversity [43,44]. Dietary intake of contaminated food is a major route of human exposure to OCPs [45,46]. This study assessed the health risks associated with adult consumption of Oviductus Ranae from Heilongjiang Province based on OCPs residue data, using estimated daily intake (EDI) and target hazard quotient (THQ) models. The EDI results showed that the daily intake of each OCP compound was far below the oral reference dose (RfDo) recommended by the United States Environmental Protection Agency (USEPA). The THQ values for all compound groups were substantially less than 1, and the total target hazard quotient (TTHQ) ranged from 0.006 to 0.020, significantly below the risk threshold of 1 (Table 2). This indicates that, at current intake levels, consuming Oviductus Ranae from Heilongjiang does not pose a significant non-carcinogenic health risk to adults. It should be noted that the risk assessment was conducted using the average daily consumption rate for general aquatic products in China (3 g/day). Given the dual medicinal and edible nature of Oviductus Ranae, its actual consumption patterns (e.g., higher intake during therapeutic periods or long-term, lower-dose use) may differ, introducing uncertainty into the exposure estimation. Therefore, future risk assessments would benefit from more product-specific consumption data.

Heptachlor epoxide contributed the highest THQ values among individual compounds (ranging from 0.00361 to 0.01768 in areas like Wuchang, Tonghe, and Hebei), followed by endosulfans and aldrin. Although its absolute EDI was low, its extremely low RfDo (0.013 μg·kg^−1^·d^−1^) resulted in a significantly higher risk quotient compared to other OCPs. This finding suggests the need for continued attention to the background residue levels and accumulation trends of such persistent pollutants in the frog’s habitat. The residue levels and corresponding THQ values for ∑HCHs and ∑DDTs were generally low, indicating that the residual impact of these historically used pesticides in the current frog farming environment is relatively limited.

Despite the overall low health risk, given the medicinal and potential long-term consumption nature of Oviductus Ranae, continuous monitoring of OCPs pollution in frog habitats is recommended, especially in areas with high industrial influence or historical pesticide use. Furthermore, considering the metabolic characteristics of pesticides like endosulfan, future studies should focus on the residue status and chronic cumulative effects of their transformation products (e.g., endosulfan sulfate) to enable a more comprehensive assessment of long-term consumption safety.

## 4. Conclusions

This study presents one of the first systematic investigation into the contamination characteristics and health risks of OCPs and PCBs in Chinese Oviductus Ranae. The results indicate that endosulfans and their metabolites are the predominant contaminants, followed by DDTs and HCHs, while the pollution burden of PCBs is relatively low. Through PCA and correlation analysis, it was revealed that the accumulation of POPs in organisms exhibits significant regional dependence, isomer selectivity, and metabolic transformation characteristics, despite variations in exposure pathways and contamination patterns. While all detected pollutant residues comply with regulatory limits, a moderate consumption frequency of Oviductus Ranae is advisable. This precautionary recommendation accounts for the cumulative nature of persistent organic pollutants (POPs), uncertainties in long-term exposure assessment, and their potential for bioaccumulation. Further research into the physiological mechanisms underlying the translocation and transformation of characteristic POPs in *Rana dybowskii* will provide a critical scientific foundation for dietary exposure risk assessment and the safe utilization of such traditional medicinal-edible products.

## Figures and Tables

**Table 1:** Levels of 17 OCPs and 9 PCBs in Oviductus Ranae from Heilongjiang Province.

Compound Name	Range (ng/g)	Mean (ng/g)	Detection Rate (%)	Limit Standards (ng/g)
α-HCH	ND—5.94 × 10^−2^	1.10 × 10^−2^	27.8	200 ^a^
β-HCH	ND—3.41 × 10^−1^	8.88 × 10^−2^	94.4	200 ^a^
γ-HCH	ND—2.92 × 10^−1^	4.11 × 10^−2^	33.3	200 ^a^
δ-HCH	ND—2.21 × 10^−1^	7.43 × 10^−2^	88.9	200 ^a^
∑HCHs	4.23 × 10^−2^–7.18 × 10^−1^	2.15 × 10^−1^	100	
o,p′-DDT	ND	ND	ND	400 ^a^
p,p′-DDD	3.44 × 10^−2^–6.46	5.25 × 10^−1^	100	400 ^a^
p,p′-DDE	ND—1.43 × 10^1^	2.38	55.6	400 ^a^
p,p′-DDT	ND	ND	ND	400 ^a^
∑DDTs	3.44 × 10^−2^–1.93 × 10^1^	2.91	100	
Heptachlor	5.18 × 10^−1^–2.59	1.22	100	
Heptachlor-epoxide	5.63 × 10^−1^–6.05	2.75	100	
∑Heptachlors	1.29–7.62	3.97	100	
α-Endosulfan	4.82 × 10^−1^–4.32 × 10^1^	1.23 × 101	100	
β-Endosulfan	1.29–2.78 × 10^1^	6.88	100	
Endosulfans sulfate	8.38 × 10^−2^–2.39	8.98 × 10−1	100	
∑Endosulfans	2.77–5.25 × 10^1^	2.01 × 101	100	
Methoxychlor	ND—1.77 × 10^1^	5.75	94.4	
Aldrin	1.25 × 10^−1^–5.26	1.31	100	
Dieldrin	ND—1.83	3.33 × 10−1	66.7	
Endrin	ND—2.34 × 10^1^	2.32	83.3	
∑DRINs	1.44–3.01 × 10^1^	9.71	100	
∑OCPs	1.17 × 10^1^–6.79 × 10^1^	3.69 × 101	100	
PCB1	7.73 × 10^−1^–4.38	1.52	100	
PCB28	ND	ND	ND	
PCB29	3.59–6.33	4.33	100	
PCB 52	ND	ND	ND	
PCB101	ND	ND	ND	200 ^b^
PCB118	ND	ND	ND	200 ^b^
PCB138	ND	ND	ND	
PCB153	ND	ND	ND	
PCB180	ND	ND	ND	
∑PCBs	4.43–8.06	5.85	100	200 ^b^

^a^: GB/T 19507—2008; ^b^: GB 2762—2022.

**Table 2:** Estimated daily intakes (*EDI*) and Target hazard quotient (*THQ*) of OCPs due to Oviductus Ranae consumption.

Compound	RfDo/µg kg^−1^·d^−1^	Acheng		Wuchang		Tonghe		Tieli		Hebei		Hulin		Heilongjiang Province	
		EDI	THQ	EDI	THQ	EDI	THQ	EDI	THQ	EDI	THQ	EDI	THQ	EDI	THQ
∑HCHs	0.3	0.00001	0.00002	0.00002	0.00008	0.00000	0.00001	0.00001	0.00003	0.00001	0.00002	0.00002	0.00005	0.00001	0.00003
∑DDTs	0.5	0.00009	0.00018	0.00028	0.00056	0.00032	0.00064	0.00008	0.00016	0.00002	0.00003	0.00006	0.00011	0.00014	0.00028
Heptachlor	0.5	0.00006	0.00011	0.00007	0.00015	0.00004	0.00009	0.00005	0.00010	0.00004	0.00007	0.00010	0.00020	0.00006	0.00012
Heptachlor-epoxide	0.013	0.00011	0.00815	0.00023	0.01768	0.00011	0.00860	0.00005	0.00361	0.00017	0.01289	0.00014	0.01076	0.00013	0.01028
∑Endosulfans	6	0.00191	0.00032	0.00214	0.00036	0.00081	0.00013	0.00041	0.00007	0.00021	0.00004	0.00038	0.00006	0.00098	0.00016
Aldrin	0.03	0.00003	0.00085	0.00002	0.00055	0.00014	0.00460	0.00003	0.00086	0.00012	0.00403	0.00005	0.00181	0.00006	0.00212
Dieldrin	0.05	0.00000	0.00001	0.00002	0.00042	0.00001	0.00028	0.00003	0.00061	0.00003	0.00052	0.00000	0.00009	0.00002	0.00032
Endrin	0.3	0.00005	0.00018	0.00002	0.00008	0.00042	0.00139	0.00007	0.00023	0.00003	0.00012	0.00007	0.00024	0.00011	0.00038
Methoxychlor	5	0.00019	0.00004	0.00021	0.00004	0.00020	0.00004	0.00024	0.00005	0.00023	0.00005	0.00059	0.00012	0.00028	0.00006
TTHQ			0.010		0.020		0.016		0.006		0.018		0.013		0.014

RfDo: oral reference dose of elements as established by the USEPA. (Retrieved 1 May 2025 from: https://iris.epa.gov/AtoZ/?list_type=alpha).

## Data Availability

The original contributions presented in this study are included in the article/Supplementary Materials. Further inquiries can be directed to the corresponding authors.

## Supplementary Materials

The following supporting information can be downloaded at: https://www.mdpi.com/article/10.3390/toxics14010101/s1, Table S1. Mass parameters, linear ranges, correlation coefficients and limits of detection (LOD) and recovery rates for the target OCPs and PCBs compounds. (*n* = 7); Table S2. Content levels of 17 OCPs and 9 PCBs in Oviductus Ranae from Heilongjiang Province; Table S3 [20,21]. Pollution index (*Pi*) for Oviductus Ranae from Heilongjiang Province; Figure S1. Sampling regions of Oviductus Ranae in the Heilongjiang Province (*n* = 54).
