# Supplementary material for: Pollution Characteristics, Sources, and Health Risks of Organochlorine Pesticides and Polychlorinated Biphenyls in Oviductus Ranae from Northern China
Source: Toxics. 2026 Jan 21;14(1):101. doi: 10.3390/toxics14010101 (PMC12846295; doi:10.3390/toxics14010101)
Supplement: Supplementary file 1 [file toxics-14-00101-s001.zip › toxics-4083869-supplementary.pdf]

Table captions

Table S1. Mass parameters, linear ranges， correlation coefficients and limits of detection (LOD) and recovery rates for the target OCPs and PCBs compounds. (*n*=7)

Table S2. Content levels of 17 OCPs and 9 PCBs in Oviductus Ranae from Heilongjiang Province.

Table S3. Pollution index (P_i) for Oviductus Ranae from Heilongjiang Province.

Table S1. Mass parameters, linear ranges， correlation coefficients and limits of detection (LOD) and recovery rates for the target OCPs and PCBs compounds. (*n*=7).

**Compound Name**	**CAS No.**	**RT (min)**	**Quantitative Ion Pair (m/z)**	**Qualitative Ion Pair (m/z)**	**Linear Range (μg/L)**	**R^2**	**LOD(μg/kg)**	**Recovery (%)**
PCB1	2051-60-7	9.26	188.0 > 152.0	190.0 > 154.0	1 - 100	0.9992	0.05	105
α-HCH	319-84-6	12.96	181.0 > 145.0	218.9 > 183.0	1 - 100	0.9992	0.44	92.3
β-HCH	319-85-7	13.47	181.0 > 145.0	218.9 > 183.0	1 - 100	0.9991	0.50	91.5
γ-HCH	58-89-9	14.72	181.0 > 145.0	218.9 > 183.0	1 - 100	0.9991	0.11	90.4
δ-HCH	319-86-8	16.53	181.0 > 145.0	218.9 > 183.0	1 - 100	0.9982	0.51	95.7
Heptachlor	76-44-8	17.62	271.8 > 236.8	273.8 > 238.8	1 - 100	0.9997	0.15	96.2
Aldrin	309-00-2	17.81	262.9 > 192.9	264.9 > 228.9	1 - 100	0.9992	0.12	89.7
Heptachlor-epoxide	1024-57-3	18.08	352.8 > 262.8	354.8 > 264.8	1 - 100	0.9993	0.48	94.4
PCB29	5103-71-9	18.29	372.0 > 266.0	374.0 > 268.0	1 - 100	0.9999	0.16	102
α-Endosulfan	959-98-8	18.75	241.0 > 206.0	339.0 > 265.0	1 - 100	0.9995	0.27	93.5
4,4'-DDE	72-55-9	20.71	246.0 > 176.0	317.9 > 246.0	1 - 100	0.9988	0.29	99.2
Dieldrin	60-57-1	22.35	277.0 > 241.0	380.8 > 345.8	1 - 100	0.9997	0.12	102
Endrin	72-20-8	24.43	263.0 > 193.0	280.9 > 244.9	1 - 100	0.9997	0.29	105
β-Endosulfan	33213-65-9	24.62	241.0 > 206.0	339.0 > 265.0	1 - 100	0.9984	0.19	92.8
2,4'-DDT	789-02-6	25.76	235.0 > 165.0	237.0 > 199.0	1 - 100	0.9995	0.47	92.7
4,4'-DDD	72-54-8	25.91	235.0 > 165.0	237.0 > 199.0	1 - 100	0.9985	0.03	99.5
Endosulfan sulfate	1031-07-8	26.4	272.0 > 237.0	387.0 > 289.0	1 - 100	0.9995	0.06	108
4,4'-DDT	50-29-3	26.62	235.0 > 165.0	237.0 > 199.0	1 - 100	0.9985	0.37	87.6
Methoxychlor	72-43-5	27.92	227.1 > 169.1	274.1 > 239.1	1 - 100	0.9997	0.31	93.1
PCB 28	7012-37-5	28.05	256.0 > 186.0	258.0 > 188.0	1 - 100	0.9990	0.01	95.5
PCB 52	35693-99-3	29.15	291.9 > 222.0	289.9 > 219.9	1 - 100	0.9988	0.06	98.7
PCB 101	37680-73-2	29.74	325.9 > 255.9	323.9 > 253.9	1 - 100	0.9992	0.16	89.3
PCB 118	31508-00-6	30.56	325.9 > 255.9	323.9 > 253.9	1 - 100	0.9980	0.30	102

PCB 138	35065-28-2	30.97	359.9 > 289.9	357.9 > 287.9	1 - 100	0.9995	0.04	96.4
PCB 153	35065-27-1	32.44	359.9 > 289.9	357.9 > 287.9	1 - 100	0.9995	0.07	107
PCB 180	35065-29-3	32.86	393.8 > 323.8	395.8 > 325.8	1 - 100	0.9994	0.15	93.9

Table S2. Content levels of 17 OCPs and 9 PCBs in Oviductus Ranae from Heilongjiang Province (μg/kg, dry weight, *n*=3).

**Compound**	**Acheng**		**Wuchang**		**Tonghe**		**Tieli**		**Hebei**		**Hulin**		**Total**		**DR**
**Name**	**range**	**mean**	**range**	**mean**	**range**	**mean**	**range**	**mean**	**range**	**mean**	**range**	**mean**	**range**	**mean**	**（%）**
α-HCH	ND - 3.71×10^{-2}	1.24×10^{-2}	ND	ND	ND	ND	ND - 5.94×10^{-2}	1.98×10^{-2}	ND - 2.53×10^{-2}	8.43×10^{-3}	ND - 5.10×10^{-2}	2.54×10^{-2}	ND - 5.94×10^{-2}	1.10×10^{-2}	27.8
β-HCH	ND - 5.16×10^{-2}	3.13×10^{-2}	1.42×10^{-1} - 3.41×10^{-1}	2.29×10^{-1}	3.62×10^{-2} - 4.65×10^{-2}	4.21×10^{-2}	4.62×10^{-2} - 6.31×10^{-2}	5.51×10^{-2}	5.95×10^{-2} - 8.06×10^{-2}	6.77×10^{-2}	5.43×10^{-2} - 1.90×10^{-1}	1.08×10^{-1}	ND - 3.41×10^{-1}	8.88×10^{-2}	94.4
γ-HCH	ND	ND	9.29×10^{-2} - 2.92×10^{-1}	1.79×10^{-1}	ND	ND	ND	ND	ND - 3.14×10^{-2}	1.05×10^{-2}	ND - 1.41×10^{-1}	5.68×10^{-2}	ND - 2.92×10^{-1}	4.11×10^{-2}	33.3
δ-HCH	ND - 1.39×10^{-1}	7.21×10^{-2}	4.79×10^{-2} - 8.48×10^{-2}	6.51×10^{-2}	2.66×10^{-2} - 8.17×10^{-2}	4.94×10^{-2}	ND - 2.21×10^{-1}	9.03×10^{-2}	2.55×10^{-2} - 9.45×10^{-2}	4.88×10^{-2}	7.52×10^{-2} - 1.91×10^{-1}	1.20×10^{-1}	ND - 2.21×10^{-1}	7.43×10^{-2}	88.9
**∑HCHs**	4.23×10^{-2} - 2.28×10^{-1}	1.16×10^{-1}	2.98×10^{-1} - 7.18×10^{-1}	4.73×10^{-1}	7.32×10^{-2} - 1.25×10^{-1}	9.15×10^{-2}	4.62×10^{-2} - 3.37×10^{-1}	1.65×10^{-1}	8.86×10^{-2} - 1.79×10^{-1}	1.35×10^{-1}	1.84×10^{-1} - 4.50×10^{-1}	3.10×10^{-1}	4.23×10^{-2} - 7.18×10^{-1}	2.15×10^{-1}	100
2,4′-DDT	ND	ND	ND	ND	ND	ND	ND	ND	ND	ND	ND	ND	ND	ND	ND
4,4′-DDD	6.32×10^{-2} - 8.41×10^{-2}	7.48×10^{-2}	5.23×10^{-2} - 2.16×10^{-1}	1.07×10^{-1}	3.44x10-2 - 6.46	2.33	3.58×10^{-2} - 9.11×10^{-2}	5.82×10^{-2}	3.47×10^{-2} - 2.45×10^{-1}	1.32×10^{-1}	7.40×10^{-2} - 1.06	4.47×10^{-1}	3.44×10^{-2} - 6.46	5.25×10^{-1}	100
4,4′-DDE	ND - 2.73	1.79	ND - 1.43×10^{1}	5.7	ND -1.29×10^{1}	4.29	ND - 3.83	1.62	ND - 6.14×10^{-1}	2.05×10^{-1}	ND - 1.27	6.88×10^{-1}	ND - 1.43×10^{1}	2.38	55.6
4,4′-DDT	ND	ND	ND	ND	ND	ND	ND	ND	ND	ND	ND	ND	ND	ND	ND
**∑DDTs**	8.41×10^{-2} - 2.79	1.86	2.16×10^{-1} - 1.44×10^{1}	5.8	3.44×10^{-2} - 1.93×10^{1}	6.63	4.76×10^{-2} - 3.87	1.68	3.47×10^{-2} - 7.30×10^{-1}	3.36×10^{-1}	2.06×10^{-1} - 2.33	1.14	3.44×10^{-2} - 1.93×10^{1}	2.91	100
Heptachlor	8.94×10^{-1} - 1.54	1.14	6.23×10^{-1} - 2.52	1.5	5.40×10^{-1} - 1.18	9.21×10^{-1}	5.18×10^{-1} - 1.87	9.94×10^{-1}	6.03×10^{-1} - 9.18×10^{-1}	7.42×10^{-1}	1.52 - 2.59	2.02	5.18×10^{-1} - 2.59	1.22	100
Heptachlor-epoxide	6.59×10^{-1} - 4.64	2.18	4.16 - 5.11	4.73	1.58 - 2.68	2.3	5.63×10^{-1} - 1.56	9.66×10^{-1}	7.60×10^{-1} - 6.05	3.45	1.51 - 4.06	2.88	5.63×10^{-1} - 6.05	2.75	100

**∑Heptachlors**	2.20 - 5.54	3.32	4.79 - 7.62	6.23	2.75 - 3.68	3.22	1.29 - 2.44	1.96	1.36 - 6.76	4.19	3.44 - 5.65	4.89	1.29 - 7.62	3.97	100
α-Endosulfan	1.46×10^{1} - 4.32×10^{1}	2.99×10^{1}	2.45×10^{1} - 3.59×10^{1}	2.84×10^{1}	4.89 - 9.96	8.24	4.84×10^{-1} - 2.27	1.39	4.82×10^{-1} - 4.34	1.86	2.02 - 6.04	4.45	4.82×10^{-1} - 4.32×10^{1}	1.23×10^{1}	100
β-Endosulfan	7.75 - 9.19	8.3	7.75 - 2.78×10^{1}	1.55×10^{1}	5.21- 8.84	7.62	3.31 - 9.71	5.84	1.29 - 2.21	1.7	1.41 - 3.18	2.31	1.29 - 2.78×10^{1}	6.88	100
Endosulfans sulfate	2.84×10^{-1} - 2.14	1.11	1.51×10^{-1} - 2.19×10^{-1}	1.93×10^{-1}	5.96×10^{-1} - 9.11×10^{-1}	7.39×10^{-1}	8.38×10^{-2} - 1.83	1.12	4.13×10^{-1} - 1.41	8.39×10^{-1}	4.89×10^{-1} - 2.39	1.38	8.38×10^{-2} - 2.39	8.98×10^{-1}	100
**∑Endosulfans**	2.48×10^{1} - 5.12×10^{1}	3.93×10^{1}	3.27×10^{1} - 5.25×10^{1}	4.41×10^{1}	1.43×10^{1} - 1.96×10^{1}	1.66×10^{1}	5.25 - 1.30×10^{1}	8.35	2.77 - 7.04	4.4	4.71 - 1.08×10^{1}	7.86	2.77 - 5.25×10^{1}	2.01×10^{1}	100
Methoxychlor	4.00×10^{-1} - 1.12×10^{1}	4.01	2.44 - 8.15	4.42	1.87 - 5.29	4.12	ND - 8.24	4.98	3.22 - 6.81	4.8	3.66 - 1.77×10^{1}	1.22×10^{1}	ND - 1.77×10^{1}	5.75	94.4
Aldrin	1.25×10^{-1} - 7.54×10^{-1}	5.23×10^{-1}	1.25×10^{-1} - 6.83×10^{-1}	3.42×10^{-1}	1.50 - 4.55	2.84	2.80×10^{-1} - 7.90×10^{-1}	5.29×10^{-1}	9.24×10^{-1} - 5.26	2.49	1.26×10^{-1} - 2.48	1.12	1.25×10^{-1} - 5.26	1.31	100
Dieldrin	ND - 3.79×10^{-2}	1.26×10^{-2}	2.53×10^{-2} - 1.19	4.36×10^{-1}	ND - 8.57×10^{-1}	2.86×10^{-1}	3.22×10^{-2} - 1.83	6.32×10^{-1}	ND - 1.53	5.40×10^{-1}	ND - 2.48×10^{-1}	9.11×10^{-2}	ND - 1.83	3.33×10^{-1}	66.7
Endrin	2.24×10^{-1} - 2.19	1.13	1.24×10^{-1} - 1.14	5.03×10^{-1}	5.12×10^{-1} - 2.34×10^{1}	8.61	3.56×10^{-1} - 3.27	1.44	ND - 2.14	7.14×10^{-1}	ND - 3.38	1.5	ND - 2.34×10^{1}	2.32	83.3
**∑DRINs**	1.44 - 1.23×10^{1}	5.67	3.47 - 9.68	5.7	7.79 - 3.01×10^{1}	1.59×10^{1}	4.09 - 9.40	7.58	6.83 - 1.02×10^{1}	8.55	5.15 - 2.19×10^{1}	1.49×10^{1}	1.44 - 3.01×10^{1}	9.71	100
**∑OCPs**	4.20×10^{1} - 5.69×10^{1}	5.02×10^{1}	5.85×10^{1} - 6.79×10^{1}	6.23×10^{1}	3.22×10^{1} - 4.87×10^{1}	4.24×10^{1}	1.86×10^{1} - 2.07×10^{1}	1.97×10^{1}	1.17×10^{1} - 2.09×10^{1}	1.76×10^{1}	2.00×10^{1} - 3.67×10^{1}	2.91×10^{1}	1.17×10^{1} - 6.79×10^{1}	3.69×10^{1}	100
PCB1	7.73×10^{-1} - 3.56	2.1	1.58 - 4.38	2.86	8.05×10^{-1} - 8.42×10^{-1}	8.23×10^{-1}	1.06 - 1.70	1.32	7.83×10^{-1} - 9.80×10^{-1}	8.86×10^{-1}	7.81×10^{-1} - 1.68	1.14	7.73×10^{-1} - 4.38	1.52	100
PCB29	3.72 - 4.50	3.98	3.69 - 4.29	4.01	3.59 - 6.33	4.79	3.89 - 5.22	4.6	3.82 - 5.23	4.54	3.71 - 4.55	4.03	3.59 - 6.33	4.33	100
PCBs*	ND	ND	ND	ND	ND	ND	ND	ND	ND	ND	ND	ND	ND	ND	ND
**∑PCBs**	4.49 - 8.06	6.09	5.87 - 8.06	6.87	4.43 - 7.17	5.62	5.09 - 6.92	5.93	4.60 - 6.21	5.42	4.61 - 6.24	5.17	4.43 - 8.06	5.85	100

Note: DR, Detection Rate (%);

ND, Not Detected;

*,7 indicator PCBs: PCB28, PCB52, PCB101, PCB118, PCB138, PCB153, PCB180.

Table S3. Pollution index (P_i) for Oviductus Ranae from Heilongjiang Province.

Elements	Limit Standards (μg/kg)	A cheng		Wu chang		Tong he		Tie li		He bei		Hu lin	
		Pi	**Pollution Level**	***Pi***	**Pollution Level**	***Pi***	**Pollution Level**	***Pi***	**Pollution Level**	***Pi***	**Pollution Level**	***Pi***	**Pollution Level**
α-HCH	200[a]	0.00006	Clean	0	Clean	0	Clean	0.00001	Clean	0.00004	Clean	0.00013	Clean
β-HCH	200[a]	0.00016	Clean	0.00114	Clean	0.00021	Clean	0.00028	Clean	0.00034	Clean	0.00054	Clean
γ-HCH	200[a]	0	Clean	0.00090	Clean	0	Clean	0	Clean	0.00005	Clean	0.00028	Clean
δ-HCH	200[a]	0.00036	Clean	0.00032	Clean	0.00025	Clean	0.00045	Clean	0.00024	Clean	0.00060	Clean
2,4′-DDT	400[a]	0	Clean	0	Clean	0	Clean	0	Clean	0	Clean	0	Clean
4,4′-DDD	400[a]	0.00019	Clean	0.00027	Clean	0.00582	Clean	0.00015	Clean	0.00033	Clean	0.00112	Clean
4,4′-DDE	400[a]	0.00448	Clean	0.01425	Clean	0.01072	Clean	0.00405	Clean	0.00051	Clean	0.00172	Clean
4,4′-DDT	400[a]	0	Clean	0	Clean	0	Clean	0	Clean	0	Clean	0	Clean
**∑PCBs**	200[b]	0.01218	Clean	0.01374	Clean	0.01124	Clean	0.01186	Clean	0.01084	Clean	0.01034	Clean

[a]: GB/T 19507 - 2008; [b]: GB 2762 - 2022.

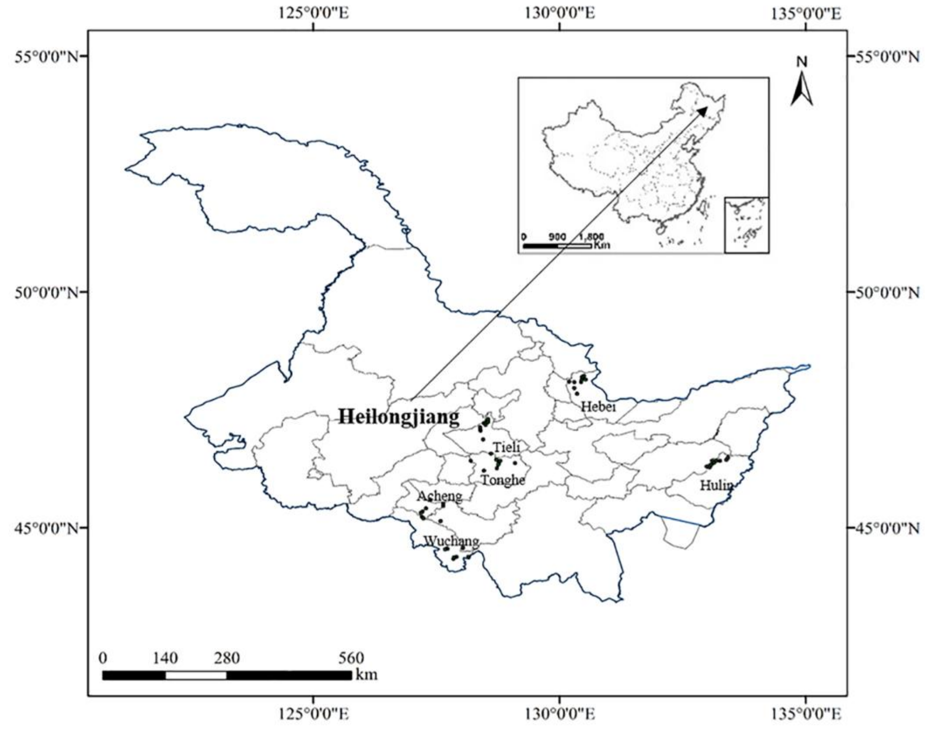

Province	Region	Longitude(E)	Latitude(N)	Number of samples
Heilongjiang	Acheng	127.19-127.71	45.14-45.59	9
Heilongjiang	Wuchang	127.68-128.15	44.34-44.57	9
Heilongjiang	Tonghe	128.20-129.10	46.21-46.57	9
Heilongjiang	Tieli	128.39-128.63	46.87-47.30	9
Heilongjiang	Hebei	130.20-130.53	47.84-48.22	9
Heilongjiang	Hulin	132.99-132.60	46.28-46.60	9

Figure S1. Sampling regions of Oviductus Ranae in the Heilongjiang Province (*n* = 54).
